# The Impact of Prenatal Diagnosis in the Evolution of Newborns with Congenital Heart Disease

**DOI:** 10.2478/jccm-2023-0007

**Published:** 2023-02-08

**Authors:** Daniela Toma, Elena Moldovan, Liliana Gozar

**Affiliations:** 1 Department of Pediatric Cardiology, Emergency Institute for Cardiovascular Diseases and Transplantation, Targu Mures, Romania; 2 Department of Pediatrics, George Emil Palade University of Medicine, Pharmacy, Science, and Technology of Targu Mures, Targu Mures, Romania; 3 Pediatric Intensive Care Unit, Emergency Institute for Cardiovascular Diseases and Transplantation, Targu Mures, Romania

**Keywords:** congenital heart malformations, prenatal diagnosis, fetal echocardiography

## Abstract

Congenital heart malformations are cardiac and/or vascular structural abnormalities that appear before birth, the majority of which can be detected prenatally. The latest data from the literature were reviewed, with reference to the degree of prenatal diagnosis regarding congenital heart malformations, as well as its impact on the preoperative evolution and implicitly on mortality. Studies with a significant number of enrolled patients were included in the research. Prenatal congenital heart malformations detection rates were different, depending on the period in which the study took place, the level of the medical center, as well as on the size of enrolled groups. Prenatal diagnosis in critical malformations such as hypoplastic left heart syndrome, transposition of great arteries and totally aberrant pulmonary venous drainage has proven its usefulness, allowing an early surgical intervention, thus ensuring improved neurological development, increasing the survival rate and decreasing the rate of subsequent complications. Sharing the experience and results obtained by each individual therapeutic center will definitely lead to drawing clear conclusions regarding the clinical contribution of congenital heart malformations prenatal detection.

## Introduction

Congenital heart malformations are cardiac and/or vascular structural abnormalities that appear before birth, the majority of which can be detected prenatally. Having an incidence between 0.8 and 1.2%, congenital heart disease (CHD) is the most frequent malformative pathology diagnosed at birth and the major cause of death in the first year of life [1,2]. In the past years, the incidence has remained somewhat constant, thus justifying the need to improve screening programs, as well as highlighting the importance of etiological causes detection.

Although it is the most diagnosed malformative pathology, the etiology of CHD is less understood [3]. In most cases, a definite cause that could have determined the appearance of the structural cardiac defect cannot be identified. Only about 10-15% of cases have a clear origin, while family aggregation, exposure to teratogens, folic acid and vitamin deficiency, obesity or pregestational diabetes are maternal conditions that are associated with an increased incidence of congenital heart malformations [4,5]. A special importance is given to the genetic causes involved in the etiology of congenital heart malformations. Till now, more than 400 specific genes have been described, which encode various transcription factors or structural proteins involved in cardiogenesis [6].

From the etiological point of view, certain environmental causes were identified that led to the increase in the incidence of heart malformations. Thus, after the Chernobyl incident in 1986, there was an increase in CHD cases. The same tendency was observed after the Fukushima nuclear accident [7].

Along with prematurity specific pathology, congenital heart malformations are the most frequent causes of hospitalization in neonatal intensive care units. The clinical differentiation between cardiogenic and septic shock is difficult in newborns. Therefore in the event of clinical signs of shock the presence of a severe cardiac structural defect must be excluded using imaging techniques such as ecochardiography. The sepsis rate in these patients may increase when a critical heart malformation is confirmed becouse of the necessary therapeutic procedures (surgical correction, extracorporeal circulation, ECMO), central vascular approach, immunosuppression, prematurity, and possibly associated extracardiac malformative pathology.

Newborns diagnosed with critical heart malformation need prolonged supervision in the intensive care unit, both in the preoperative period, but especially in the postoperative period. There is a clear correlation between the length of hospitalization in the intensive care unit and the mortality rate in the first year of life. The main cause of heart failure in newborns is the presence of a critical congenital malformation at this level. Newborns with congenital heart malformation who develop severe heart failure, pulmonary edema, severe arrhythmias or any other condition that endangers the cardiac status require intensive care therapeutic measures. In case of a critical heart malformation prenatal diagnosis it is recommended for the birth to take place in a level 3 medical center with a Neonatal Intensive Care Service. The therapeutic measures in the intensive care service are aimed at ensuring adequate tissue perfusion and obtaining optimal oxygenation.

## Material and method

The aim of this article was to corroborate the latest data from the literature regarding the influence of prenatal diagnosis on mortality and preoperative complications of the newborn with congenital heart disease. The latest data from the literature were reviewed, with reference to the degree of prenatal diagnosis regarding congenital heart malformations, as well as its impact on the preoperative evolution and implicitly on mortality. Studies with a significant number of enrolled patients were included in the research.

## Results

Prenatal CHD detection rates were different, depending on the period in which the study took place, the level of the medical center, as well as on the size of enrolled groups. Prenatal diagnosis of heart malformations may increase the survival rate and decrease the rate of subsequent complications. Ensuring the birth takes place in a medical center with neonatal intensive care unit, cardiac surgery and interventional cardiology services becomes crucial in the management of cardiac malformations. Thus, the risk of hemodynamic instability in the early neonatal period is significantly reduced [8]. (Table 1).

**Table 1:** Influence of prenatal diagnosis on mortality and preoperative complications of the newborns with congenital heart disease – data from reviewed studies

**Author**	**Study period**	**Study design**	**Prenatal detection rate**	**Morbidity**	**Mortality**
Chakraborty et al. [9]	10 years	retrospective	68,1 %	↓antibiotic therapy, inotropic need, liver/kidney dysfunction, acidosis	uninfluenced
van Velzen et al. [10]	10 years	prospective/ retrospective	59,7%	not mentioned	not mentioned
Gorla et al. [11]	18 years	retrospective	68,8%	not mentioned	uninfluenced
Gupta et al. [2]	5 years	retrospective	62 %	low doses of PGE low doses of iNO	uninfluenced
Suard et al. [12]	4 years	retrospective	71,5%	↑ duration of hospitalization in PICU	uninfluenced

PGE- Prostagladin E; iNO- inhaled nitric oxid; PICU- Pediatric Intensive Care Unit

## Discussions

Prenatal diagnosis of congenital heart malformations has become a routine examination among high-risk mothers, the detection rate being variable depending on the evaluation center [8,13]. The benefits of prenatal diagnosis on morbidity and mortality of the newborn with congenital heart malformation are debatable. The redefinition of screening indications and continuous technical progress have led to an increase in the degree of early prenatal detection of congenital heart malformations [11]. Fetal echocardiography must be performed by trained personnel and interpreted according to fetal cardiac anatomy and physiology. This allows for an understanding in dynamics of the heart physiology and offers the possibility of evaluating the necessity of fetal or neonatal treatment [8]. The moment of diagnosis is extremely important in the evolution of the pregnancy. Measurement of the nuchal translucency, tricuspid regurgitation detection or changes in the ductus venosus flow during the ultrasound examination from weeks 11-13 of pregnancy can lead to an early diagnosis of congenital heart disease [14].

According to Sun et al. fetal echocardiography is recommended to be performed in the following maternal conditions: gestational diabetes or pregestational diabetes, lupus or Sjogren’s syndrome with positive serology (anti-SSA/anti-SSB antibodies), obtaining pregnancy through assisted reproduction techniques, viral infection with the rubeola virus, Coxsackie or Parvovirus, uncontrolled phenylketonuria, as well as in the case of a first-degree relative with congenital heart disease. There are also a series of fetal conditions that require a fetal echocardiography. Raising the suspicion of a structural heart defect during routine obstetric ultrasound, detecting a fetal arrhythmia, identifying an extracardiac malformation or a chromosomal defect require additional investigations. Nuchal translucency over 3 mm, fetal hydrops, monochorionic twin pregnancies or placental malformations are situations in which a fetal echocardiography must be performed [5,8].

The prenatal detection of a congenital heart malformation must be followed by the identification of other possible associated anomalies, and thus providing more time to set treatment strategies and to better understand the prognosis of these still unborn babies, but at the moment the detection rate is variable [8,11,15]. Understanding the fetal cardiac anatomy and physiology has led to the improvement of prenatal and perinatal management, with a decrease in associated morbidity [5].

The rate of prenatal detection of congenital heart malformations has improved significantly in recent decades. Van Velzen et al. conducted a cohort study over a period of ten years, carried out in three third-level centers, including over 700 newborns. They tracked the degree of accuracy between prenatal and postnatal diagnosis. Over 80% of the subjects had an anatomically correct diagnosis [10].

There are studies in which it was not possible to demonstrate the decrease in mortality among newborns with a prenatal diagnosis of congenital heart malformation [11,15]. This is proven by the study developed by Chakraborty et al. in which the mortality rate was not influenced by the prenatal detection rate of the cardiac malformation, but there was an improvement in the preoperative morbidity among newborns with ductal-dependent heart malformations. Prenatal diagnosis of hypoplastic left heart syndrome, transposition of large vessels or coarctation of the aorta led to a decrease in postnatal morbidity and mortality [15].

Chakraborty et al. demonstrated the fact that the lack of prenatal diagnosis is more frequently associated with acidosis, considering the late initiation of Prostaglandin E1 therapy in ductal-dependent cardiac malformations. The lack of prenatal diagnosis and the onset of sepsis-like clinical signs lead to the more frequent use of antibiotics among these patients. An increase in the survival rate in ductal-dependent cardiac malformations with prenatal diagnosis was also observed [9].

The retrospective research carried out by Gupta et al. over a period of 5 years included approximately 300 newborns, with a chronological age of less than 10 days and a gestational age of more than 34 weeks. The prenatal detection rate of congenital heart malformation was 62%. This was not influenced by sex, birth weight or gestational age. The study focused on the therapy needed during the transport to the regional pediatric cardiology center. The obtained results proved that newborns with a prenatal diagnosis of CHD required less inotropic support and mechanical ventilation during transport. In ductal-dependent congenital malformations initiation of Prostaglandin E1 therapy was carried out later, thus higher doses being necessary in the case of newborns with postnatal diagnosis. The survival rate at the age of 1 year was somewhat similar in both study groups. Antenatal diagnosis of hypoplastic left heart syndrome was the most frequent cardiac pathology. Therapeutic approach, duration of mechanical ventilation, administration of Prostaglandin E1, as well as need for inotropic support were similar in both study groups. The duration of hospitalization and the survival rate were almost equal. As well as therapeutic approach, it was found that the need for nitric oxide (NO) was significantly higher among newborns with transposition of large vessels detected postnatally [2].

In the study conducted by Gorla et al., in which over 300 patients diagnosed with complex heart malformations were enrolled, the prenatal detection rate was over 60%, with a gradual increase among conotruncal malformations [11]. A similar diagnosis rate was also reported by Chakraborty et al. in a retrospective study carried out over a period of ten years [9].

The management of the newborn with congenital heart malformation is a complex one. These patients are in need of fluid resuscitation, inotropic and respiratory support, as well as correction of arrhythmias or coagulation disorders and selective therapy for any associated infections. The administration of prostaglandin E1 in ductal-dependent congenital malformations is crucial since its role is maintaining the permeability of the ductus arteriosus until the moment of corrective surgery. Extracorporeal life support (ECMO) is part of the therapy of the critically ill patient diagnosed with congenital heart malformation, providing support when the myocardial function is temporarily impaired. Even if the prenatal detection rate has increased significantly, the results obtained regarding the mortality rate and the complexity of preoperative morbidities are contradictory. Although in some studies there was no decrease in mortality among newborns with prenatal diagnosis, among those with ductal-dependent structural defects, a decrease in morbidity was observed in the preoperative period. Thus, mechanical ventilation, antibiotic therapy or inotropic support were less often approached as therapy, in newborns with prenatal diagnosis. Liver and kidney dysfunctions, as well as acidotic metabolic disorders were less often detected in the preoperative period among those with prenatal suspicion of heart defect. [9,11]

The morbidity generated by congenital heart malformations and the influence of prenatal diagnosis on them are extremely important. Bonnet et al. demonstrated the fact that attention must be directed to the degree and severity of associated morbidities, considering that mortality is not influenced by prenatal diagnosis [16]. From his point of view, in order to decrease the severity and the rate of associated diseases, the strategies must be oriented towards fetal interventions, improving the diagnosis and ensuring an individualized postnatal management for each particular case. Interventional therapies performed during the intrauterine period are indicated in critical aortic stenosis, pulmonary atresia or hypoplastic left heart syndrome with intact interatrial septum. The type of cardiac malformation, the associated extracardiac defects, their framing in the context of a genetic syndrome, the therapeutic possibilities in the neonatal period, are conditions that weigh a lot in making the decision to continue or not the respective pregnancy. Also, family, society and religious considerations have a significant impact in making such a decision [16,17].

The study of Levey et al. was also a retrospective one, carried out over a period of 4 years, the degree of prenatal detection being 68%. The results obtained by them, regarding the benefits of prenatal diagnosis are uncertain. Their study found that ductal-dependent cardiac malformations, such as univentricular heart were more frequently detected in the prenatal period. The group with prenatal diagnosis required less invasive ventilatory support, antibiotic therapy, cardiac catheterization or corrective intervention in emergency conditions [18]. Regarding the need for invasive ventilatory support and antibiotic therapy in the preoperative period, the same results were obtained by the study conducted by Chakraborty [9]. Regarding the adaptation to extra-uterine life sustained by the Apgar Score, there were no significant differences between the two groups. Also, the preoperative metabolic status, the surgical intervention length, hospitalization duration and overall mortality were not different between those with prenatal diagnosis versus those with postnatal diagnosis [18].

Prenatal diagnosis significantly changed the timing of surgical intervention, which was carried out earlier [16,17]. Early surgical correction, especially in patients with transposition of the great arteries or hypoplastic left heart syndrome prevents the occurrence of neurological injury generated by hypoxemia and hemodynamic instability. National screening programs represent the revolution in diagnosis and management of newborns with congenital heart malformation. This is closely sustained by the results obtained by val Velzen et al., the introduction of national screening programs in the Netherlands leading to an increase of over 20% in the prenatal detection rate of congenital heart malformations [10].

The same notable results were recorded following the introduction of regional screening programs in Sweden where there was an increase in the prenatal detection rate of heart malformations from 13.8% in 2009 to 56% in 2016 [19]. Prenatal detection of congenital heart malformations correlated with maternal age, positive family history of heart malformation, maternal diabetes, twin pregnancies, as well as the complexity of the malformation or the association with other extra-cardiac structural defects. The detection rate was lower among overweight and obese patients, as well as among those with pregestational arterial hypertension [5].

The study carried out by Quartermain included over 10,000 newborns who required surgical intervention in over 100 medical centers. The results obtained showed that those with prenatal diagnosis had fewer major risk factors [17].

In the study of Suard et al., carried out over a period of 4 years, the overall prenatal detection rate was 71.5%. With regard to the diagnosed pathology, transposition of the great arteries had a detection rate of 80%, coarctations of the aorta represented a proportion of 56%, and total aberrant pulmonary venous drainage was detected in a percentage of 20%. This study also did not report any benefit of echocardiographic screening in terms of mortality [12].

Sometimes the limited acoustic window restricts the contribution of fetal echocardiography, which is why additional methods of prenatal diagnosis are needed. In borderline cases, the fetal MRI evaluation brings additional information in order to establish the subsequent therapeutic attitude, including timing and type of birth, as well as evolution in the early neonatal period [20].

The difference in results of the various studies can be partly explained by the type and length of each research, most being retrospective ones, as well as other factors such as pregnancy-associated pathologies, timing and operative technique, and neonatal intensive care unit level.

## Conclusions

Prenatal diagnosis in critical malformations such as hypoplastic left heart syndrome, transposition of great arteries and totally aberrant pulmonary venous drainage has proven its usefulness, allowing an early surgical intervention, thus ensuring improved neurological development. Sharing the experience and results obtained by each individual therapeutic center will definitely lead to drawing clear conclusions regarding the clinical contribution of congenital heart malformations prenatal detection. Even if mortality is an indicator somewhat unaffected by the degree of prenatal detection, this is still somewhat subjective, considering the fact that most critical ductal-dependent malformations with insidious evolution are now detected in the intrauterine period. The differences in reporting suggest the persistence of unequal access to medical services and the use of different protocols regarding the risk of structural heart defects in the fetus.
